# Improvement and Variability of Adolescent Backstroke Swimming Performance by Age

**DOI:** 10.3389/fspor.2020.00046

**Published:** 2020-04-23

**Authors:** Khaled Alshdokhi, Carl Petersen, Jenny Clarke

**Affiliations:** School of Health Sciences, College of Education, Health and Human Development, University of Canterbury, Christchurch, New Zealand

**Keywords:** race, progression, maturation, competitive, gender

## Abstract

To predict future performance, coaches rely on their previous experiences with a relatively small number of adolescent competitive swimmers to estimate the rate of improvement. The purpose of this study is to quantify the annual change in competition performance as backstroke swimmers mature. Data from 2006 to 2017 provided 9,956 swimming years of accumulated data which was used to estimate the rate of improvement of male and female backstroke swimmers as they aged from 8 to 18 years. Swimming performance improved rapidly between 8 and 13 years, and improvements diminished as swimmers approached their performance potential around 18 years old. These results provide accurate age-based progression data for adolescent backstroke swimmers, providing baseline performance prediction for coaches to predict future performance as swimmers mature, and providing a measure against which potential improvements from novel coaching and training methods can be objectively evaluated.

## Introduction

A swimmer's performance fluctuates over time and accurately tracking performance improvement is complicated given within and between competition performance changes, as well as whether a swimmer is in a peaking or a heavy training macrocycle. Low performance stability in adolescent swimmers is affected not only by growth and maturation but also potentially confounded by injury, illness, inappropriate training volumes and better support and training conditions (Costa et al., 2011). Despite this, coaches will often use time trial performance in training to help predict subsequent competition times, with the utility of time trial protocols to indicate performance and physiological capacities underpinned by the fact that VO_2_max is achieved during a 400 m Time Trial (Zacca et al., 2019). Yet, competition times are expected to be faster than training times. As demonstrated by Tor et al. (2014) when they compared swimming variables between three time-trials and one competition performance for 10 elite swimmers, there was a 2.4% difference between time trial and competition performance across the swimmers' different strokes.

Swimmer's times also vary between competitions as demonstrated by Stewart and Hopkins (2000) who found a typical competition-to-competition variation of 1.4% (95% likely range of true value, 1.3–1.5%) for the same stroke and event in their analysis of 532 junior and national level swimmers. Notably, faster swimmers displayed less variation (1.1%; 0.9–1.4%) than slower swimmers 1.5%; 1.3–1.9%). This parallels findings with the sport's most elite athletes: Fulton et al. (2009) calculated a between competition performance variation of ~1% with data from 242 elite Paralympic swimmers. Further, Pyne et al. (2004) calculated a between competition performance variation of 0.8%; 0.73–0.86%, with data from 51 Olympic level swimmers. Interestingly, a more recent study of 19 elite swimmers during their 8 months build up to the Rio Olympics reported variations of between ~0.7–0.5% with greater variation for sprinters compared to middle distance swimmers and males compared to females (Clephas and Wilhelm, 2019).

As evidenced above, researchers recognize the importance of quantifying competition-to-competition variability in swimming performance. This enables estimation of the smallest worthwhile performance change, which in turn helps coaches to define realistic goals and training methods (Pyne et al., 2004). For example, an improvement of ~0.4% between competitions will give swimmers a substantially increased chance of winning a medal (Hopkins et al., 1999; Hopkins, 2004). Similarly, Fulton et al. (2009) recommends that Paralympic swimmers wanting to substantially increase medal prospects need an annual improvement of at least 1–2%. In fact, factors that change performance time by as little as 0.5% will affect the placing of a top junior swimmer (Stewart and Hopkins, 2000).

Elite medal time predictions are one benefit of understanding longitudinal performance changes from competition to competition, and from season to season. Another is the estimation of the age corresponding to peak performance and the length of this peak-performance window. For example, Allen et al. (2014) calculated that Men typically peak at 24 and women at 22 years, and the window of maintaining peak-performance being ~2.6 ± 1.5 years. However, the vast majority of swimmers are more interested in knowing how their rate of improvement compares to their age and gender matched peers, especially as they mature from junior to senior competition. This information needs to be stroke specific and tracked over multiple years. The vast majority of swimming research has been reported for the freestyle stroke, with scarcity of information on the other three competitive strokes including backstroke. Therefore, the aim of our current retrospective analysis is to provide descriptive benchmark data to assess expected yearly improvement rates by pool length (course), distance and gender for adolescent backstroke swimming. Our hypothesis is that backstroke swimming will display the largest percentage improvements at the youngest ages before demonstrating a plateau at around age 14 and 16 years in females and males, respectively, in line with reaching physical maturity.

## Materials and Methods

### Procedures

The publicly available Swimming New Zealand ^1^ which can be accessed through the Swimming New Zealand website (https://swimming.org.nz/) collates data from official swimming events from club championships, inter-club, regional and international meets that Swimming New Zealand members compete in. Using this database, we retrospectively extracted and collated the official race times of backstroke events for swimmers aged 8 to 18 years over an 11-year period, from 2006 to 2017 inclusive. Data was categorized as either long or short-course (25 or 50 m pool length) and further categorized by race distance (50, 100, and 200 m) and swimmer age and gender. As we were interested in year to year performance change, swimmers needed to have swum for more than 1 consecutive year to be included in the analysis.

### Statistical Analysis

We used descriptive statistics (mean ± SD) to represent the mean performance times and likely performance change with the associated spread of values. In particular, the mean yearly percentage improvement for each age-group was calculated by comparing each swimmer's current year's best time to their fastest swim from the previous year; with the exception of a swimmer's first competitive year where their first ever swim was used as the comparison baseline. Any starting year where a swimmer only had a single swim result was excluded from our analysis. All swimmers' individual yearly performance changes were then averaged to provide the overall mean for each respective age band, course, distance and gender. As a normative stability parameter, the Pearson correlation between paired performances throughout the eleven chronological ages was determined. Here, stability was considered to be high if *r* > 0.60, and moderate if 0.30 < *r* <0.60 as suggested by Malina (2001). Data were also graphed displaying the difference between the mean (± SD) time for each age and gender with the respective fastest individual time.

## Results

The collated 11 years of data provided an accumulated total of 9,956 swimming years. At the youngest ages the longest events were the least competitive with the lowest number of competitors in both short and long-course contests (see Table 1). The mean performance times improved by approximately one third (34–42%) from age 8 to 18 years, with similar percent changes in long and short course and for each gender (see Table 2). The greatest improvements were at the younger ages, and overall there was a trend for larger performance improvements the shorter the distance. Interestingly, the female 200 m short course showed the only performance detriment (negative performance improvement), see Table 3. Normative stability values were all high and ranged between 0.66 and 0.98 for short course performance whereas long course performance ranged between 0.71 and 0.96 for long course performances, interestingly there was a trend of improved stability (>0.90) especially after age 16. Table 4, displays the number of races underpinning the data calculated in the earlier tables, and shows most swimmers had between 2 and 6 races for each respective event analyzed.

**Table 1:** Number of swimmers analyzed by course, distance and age.

	**Age (Years)**										
	**8**	**9**	**10**	**11**	**12**	**13**	**14**	**15**	**16**	**17**	**18**
**Female—Short Course**											
50 m Backstroke	77	77	75	76	76	75	75	82	75	84	49
100 m Backstroke	71	80	83	77	82	78	75	88	75	80	71
200 m Backstroke	23	79	86	82	75	77	77	80	76	80	51
**Female—Long Course**											
50 m Backstroke	70	82	75	80	80	79	81	75	85	81	67
100 m Backstroke	62	85	84	79	80	86	83	84	81	79	75
200 m Backstroke	15	71	77	79	76	77	76	76	76	86	66
**Male—Short Course**											
50 m Backstroke	79	84	86	81	79	82	83	80	89	77	64
100 m Backstroke	76	79	81	81	78	78	76	81	77	101	74
200 m Backstroke	16	76	82	76	70	79	79	75	75	77	62
**Male—Long Course**											
50 m Backstroke	62	79	87	78	80	83	80	92	77	75	65
100 m Backstroke	54	75	77	77	78	78	75	81	78	82	74
200 m Backstroke	13	72	76	75	77	76	76	75	75	78	68

**Table 2:** Backstroke mean (±SD) race time (s) by age.

	**Age (Years)**										
	**8**	**9**	**10**	**11**	**12**	**13**	**14**	**15**	**16**	**17**	**18**
**Female Short Course**											
50 m Backstroke	50.98 ± 5.72	46.77 ± 4.97	42.88 ± 5.12	39.06 ± 4.31	37.06± 4.15	35.30 ± 4.29	33.99 ± 2.89	32.64 ± 2.73	31.97 ± 2.42	32.12 ± 2.88	32.08 ± 5.26
100 m Backstroke	105.27 ± 8.80	96.54 ± 8.02	89.96 ± 9.03	83.41 ± 8.89	78.19 ± 8.63	74.43 ± 7.76	72.84 ± 7.9.8	70.50 ± 6.73	69.35 ± 6.21	69.19 ± 8.26	68.14 ± 10.04
200 m Backstroke	217.96 ± 16.16	199.53 ± 15.51	185.29 ± 14.05	173.36 ± 14.52	163.78 ± 13.82	155.93 ± 13.01	150.76 ± 13.09	148.54 ± 12.16	146.35 ± 9.86	143.84 ± 9.16	143.78 ± 10.60
**Female Long Course**											
50 m Backstroke	50.91 ± 4.64	45.90 ± 4.37	42.88 ± 4.12	40.42 ± 3.95	37.37 ± 3.29	35.59 ± 3.10	34.58 ± 2.82	33.96 ± 2.77	33.05 ± 2.20	32.69 ± 2.10	31.87 ± 4.45
100 m Backstroke	106.65 ± 9.49	97.45 ± 9.26	91.05 ± 7.61	85.06 ± 7.26	79.96 ± 7.13	75.79 ± 5.62	74.14 ± 5.69	73.11 ± 6.65	72.33 ± 5.92	70.87 ± 5.54	69.89 ± 5.20
200 m Backstroke	214.19 ± 22.73	198.82 ± 17.30	183.80 ± 12.15	174.53 ± 12.72	165.18± 12.19	159.00 ± 10.96	155.13 ± 10.94	154.48 ± 12.77	151.02 ± 10.88	149.84 ± 10.76	149.16 ± 11.10
**Male Short Course**											
50 m Backstroke	51.36 ± 6.26	46.71 ± 5.13	42.38 ± 4.01	39.94 ± 3.94	36.77 ± 3.48	34.60 ± 3.42	33.25 ± 3.00	32.05 ± 2.75	29.88 ± 2.28	29.60 ± 2.60	29.02 ± 2.36
100 m Backstroke	105.96 ± 9.29	98.04 ± 9.37	89.75 ± 7.62	84.08 ± 6.83	78.11 ± 6.15	71.88 ± 6.27	68.06 ± 5.61	65.46 ± 4.86	63.58 ± 4.89	62.16 ± 4.28	62.40 ± 6.16
200 m Backstroke	207.46 ± 15.51	199.94 ± 16.98	184.73 ± 13.49	171.34 ± 11.03	162.87 ± 11.73	153.00 ± 11.83	144.43 ± 10.58	141.99 ± 14.57	137.30 ± 14.39	133.92 ± 11.60	131.72 ± 12.76
**Male Long Course**											
50 m Backstroke	50.29 ± 6.39	45.75 ± 4.66	42.23 ± 3.81	39.67 ± 3.67	37.54 ± 3.54	35.28 ± 3.74	32.82 ± 4.42	32.05 ± 2.75	31.30 ± 2.78	30.71 ± 2.68	29.66 ± 2.02
100 m Backstroke	104.40 ± 10.04	96.32 ± 9.27	89.02 ± 7.84	84.00 ± 6.64	80.24 ± 6.96	75.21 ± 7.91	71.50 ± 6.89	68.65 ± 5.58	66.42 ± 5.38	65.50 ± 4.94	63.54 ± 4.61
200 m Backstroke	201.71 ± 16.46	194.97 ± 11.95	182.74 ± 10.16	174.62 ± 10.87	167.15 ± 13.11	156.53 ± 12.00	149.10 ± 10.89	144.45 ± 9.69	141.23 ± 9.51	137.96 ± 9.49	136.51 ± 10.49

**Table 3:** Backstroke percentage yearly performance improvement (±SD) by age, course and distance.

	**Age (Years)**										
	**8**	**9**	**10**	**11**	**12**	**13**	**14**	**15**	**16**	**17**	**18**
**Female—Short Course**											
50m Backstroke	9.6 ± 6.7	6.9 ± 6.8	8.0 ± 5.3	7.1 ± 4.6	5.8 ± 4.2	5.8 ± 3.7	3.8 ± 3.4	2.3 ± 3.1	1.2 ± 2.8	1.9 ± 3.1	0.6 ± 3.1
100m Backstroke	6.9 ± 5.7	7.7 ± 5.9	7.9 ± 4.6	6.7 ± 4.9	5.5 ± 4.6	4.1 ± 3.1	2.4 ± 3.2	2.0 ± 3.0	1.0 ± 3.3	0.9 ± 2.8	1.9 ± 5.6
200m Backstroke	2.9 ± 4.6	6.9 ± 4.5	6.4 ± 4.3	5.9 ± 4.5	5.4 ± 3.9	3.4 ± 3.7	2.6 ± 3.1	1.6 ± 2.6	0.5 ± 2.5	1.2 ± 3.5	−0.1 ± 3.1
**Female—Long Course**											
50m Backstroke	5.8 ± 4.8	8.1 ± 5.3	6.5 ± 4.9	6.2 ± 3.7	6.1 ± 3.5	3.5 ± 3.4	2.5 ± 2.4	1.2 ± 2.4	2.3 ± 2.9	0.4 ± 2.2	0.2 ± 2.6
100m Backstroke	4.5 ± 4.6	7.9 ± 4.9	6.3 ± 4.4	6.5 ± 4.1	6.0 ± 3.9	4.0 ± 3.4	2.4 ± 2.6	1.6 ± 2.6	0.5 ± 2.7	0.5 ± 3.0	0.9 ± 2.8
200m Backstroke	4.9 ± 4.4	5.4 ± 4.0	6.2 ± 4.5	5.3 ± 3.6	5.2 ± 3.6	3.4 ± 3.0	2.8 ± 3.0	0.6 ± 2.4	1.2 ± 2.3	0.4 ± 2.7	0.1 ± 2.9
**Male—Short Course**											
50m Backstroke	8.97 ± 6.1	9.2 ± 6.0	9.3 ± 5.5	6.1 ± 5.2	6.4 ± 4.8	5.9 ± 5.6	5.6 ± 4.4	4.2 ± 3.2	2.6 ± 2.4	0.9 ± 2.6	1.4 ± 4.5
100m Backstroke	7.3 ± 5.2	7.3 ± 4.9	8.0 ± 6.1	6.5 ± 4.9	6.5 ± 4.4	6.9 ± 4.7	5.0 ± 3.6	3.3 ± 3.3	1.9 ± 3.2	1.6 ± 3.0	0.1 ± 3.3
200m Backstroke	5.5 ± 6.0	5.3 ± 4.2	6.5 ± 5.1	6.6 ± 4.2	4.8 ± 4.1	4.6 ± 3.9	5.9 ± 8.5	2.5 ± 3.2	1.9 ± 3.9	1.6 ± 2.5	0.8 ± 2.7
**Male—Long Course**											
50m Backstroke	7.0 ± 5.3	6.9 ± 6.3	7.5± 5.3	5.5 ± 4.4	5.6 ± 5.0	5.2 ± 4.4	4.7 ± 3.9	3.2 ± 3.2	2.3 ± 3.3	1.4 ± 2.6	1.4 ± 2.1
100m Backstroke	4.2 ± 4.3	6.6 ± 4.5	7.6 ± 4.6	5.2 ± 4.0	5.0 ± 3.7	6.2 ± 4.6	4.5 ± 3.9	3.4 ± 2.9	2.3 ± 2.7	0.8 ± 2.7	0.8 ± 2.5
200m Backstroke	3.2 ± 5.6	4.6 ± 4.2	6.1 ± 4.0	4.4 ± 3.1	4.8 ± 4.0	4.9 ± 3.9	4.2 ± 3.4	2.7 ± 3.1	1.8 ± 2.4	1.0 ± 2.2	0.5 ± 3.1

**Table 4:** Number of races per year (±SD) for individual swimmers by age, course and distance.

	**Age (Years)**										
	**8**	**9**	**10**	**11**	**12**	**13**	**14**	**15**	**16**	**17**	**18**
**Female—Short Course**											
50 m Backstroke	5.2 ± 2.3	5.5 ± 2.6	5.4 ± 2.6	5.5 ± 2.9	6.2 ± 3.3	6.0 ± 2.7	6.3 ± 2.7	6.0 ± 2.9	5.6 ± 3.4	5.5 ± 2.4	4.7 ± 2.4
100 m Backstroke	4.2 ± 3.0	3.9 ± 2.2	3.4 ± 2.1	3.6 ±2.1	3.8 ± 2.3	4.0 ± 2.5	3.9 ± 2.9	3.8 ± 2.4	3.5 ± 2.1	4.0 ± 2.5	5.4 ± 5.2
200 m Backstroke	2.4 ± 0.7	2.8 ± 1.1	3.0 ± 2.1	3.0 ± 1.9	3.2 ± 2.2	3.7 ± 2.4	4.3 ± 2.9	4.1 ± 2.8	3.2 ± 1.8	3.5 ± 1.9	3.3 ± 2.3
**Female—Long Course**											
50 m Backstroke	3.4 ± 1.3	4.2 ± 1.6	3.7 ± 1.7	4.3 ± 1.7	4.4 ± 2.3	4.4 ± 2.8	4.8 ± 3.2	4.8 ± 2.7	4.7 ± 2.6	4.5 ± 2.8	4.6 ± 3.0
100 m Backstroke	2.9 ± 1.3	3.7 ± 1.6	3.3 ± 1.4	3.6 ± 1.7	3.4 ± 2.0	4.0 ± 3.3	4.5 ± 3.3	5.0 ± 3.5	4.5 ± 2.9	4.4 ± 2.6	4.6 ± 2.7
200 m Backstroke	2.6 ± 0.6	3.0 ± 1.1	3.4 ± 1.7	3.1 ± 1.7	3.5 ± 2.1	4.1 ± 3.0	4.8 ± 3.2	4.8 ± 3.5	4.6 ± 2.5	4.1 ± 2.4	3.9 ± 2.7
**Male—Short Course**											
50 m Backstroke	4.0 ± 1.7	3.8 ± 2.4	3.9 ± 2.4	4.0 ± 2.5	3.8 ± 2.3	3.6 ± 2.2	4.0 ± 2.4	4.0 ± 2.8	4.2 ± 2.5	3.7 ± 2.3	3.1 ± 2.1
100 m Backstroke	3.6 ± 1.6	3.8 ± 2.1	3.5 ± 2.3	3.8 ± 2.4	4.2 ± 2.8	4.6 ± 2.6	4.7 ± 3.3	4.9 ± 3.3	5.3 ± 8.4	4.1 ± 2.4	3.1 ± 2.3
200 m Backstroke	2.7 ± 1.4	2.8 ± 1.4	2.9 ± 1.9	3.0 ± 2.2	3.0 ± 2.1	3.3 ± 2.1	3.8 ± 2.6	3.5 ± 2.2	3.8 ± 2.3	3.5 ± 2.2	3.0 ± 2.1
**Male—Long Course**											
50 m Backstroke	3.9 ± 1.4	3.9 ± 1.7	3.5 ± 1.8	3.4 ± 1.8	3.1 ± 1.9	3.5 ± 2.3	3.4 ± 2.6	3.6 ± 2.5	3.5 ± 2.8	3.4 ± 2.5	4.0 ± 2.9
100 m Backstroke	3.0 ± 1.1	3.4 ± 1.8	3.7 ± 2.1	3.4 ± 1.7	3.3 ± 1.9	3.8 ± 2.4	3.8 ± 2.4	4.9 ± 2.1	4.3 ± 2.5	3.5 ± 2.7	4.3 ± 3.4
200 m Backstroke	2.5 ± 1.0	2.7 ± 1.1	2.8 ± 1.9	3.1 ± 2.2	2.9 ± 2.1	3.1 ± 2.0	3.4 ± 2.2	3.8 ± 2.3	3.8 ± 2.3	3.5 ± 2.3	2.8 ± 1.5

## Discussion

The primary result of this analysis is a benchmark for improvement as adolescent backstroke swimmers mature. This data should be valuable to coaches and parents wanting to assess the relative improvement of a given swimmer. Coaches may also use the data to highlight realistic expectations of a child's performance potential in their discussions with over-zealous parents to identify targets for distances at specific times of a swimmer's career. Statistically savvy coaches could also generate forest plots of Z-scores for swimmers to highlight each swimmer's relative strengths across different distances and course types in comparison to their age group. Monitoring the progression of athletes with regular performance tests is a useful practice, however, according to Hopkins (2004) there is also a widespread lack of understanding about the interpretation of changes in test scores, “*Perhaps the most important issue is that of magnitude: to interpret the change in an athlete's performance since a previous test, you need some concept of the magnitude of change that matters to the athlete in his or her sport*.”

Similar studies have been undertaken on Freestyle (Costa et al., 2011) and Breaststroke (Costa et al., 2010) and using this type of data coaches and swimmers can discuss the assessment of each of their strokes especially when the time comes to make a more informed decision as to their relative stroke strengths. This may also provide useful data as to the extent that certain strokes are earlier or later to develop as a swimmer matures toward their full adult potential. Intuitively, some strokes might rely relatively more on technique as opposed to absolute strength/power. Stewart and Hopkins (2000) concluded that swimmers are stroke specialists, and should concentrate training and competing on a particular stroke rather than a particular distance. However, when to specialize is a contentious topic and beyond the scope of this paper.

Predictably, there was a diminishing rate of improvement, but greater normative stability as swimmers mature and approached their performance potential. Stability of athletic performance is said to help researchers to predict future success of talented young athletes, and our data agrees with that of Costa et al. (2011) who investigating freestyle swimmers found that the ability to predict swimmer's likely adult standard is increased after 16 years.

**Figure 1:**
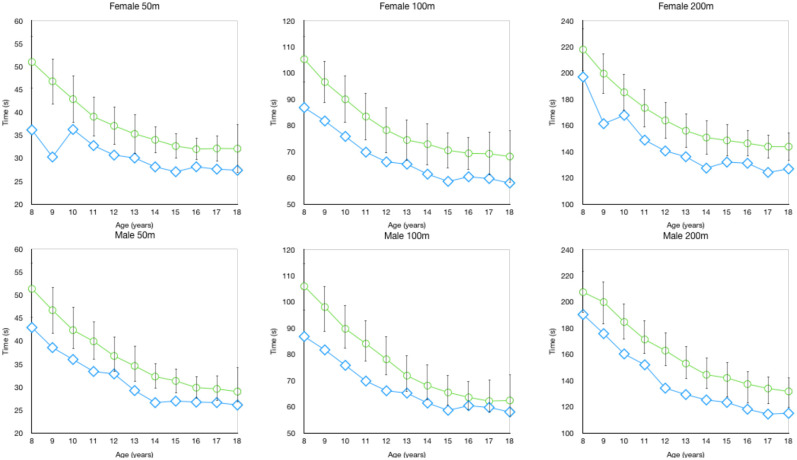
Short course backstroke mean (±SD) race time (s) compared to the overall fastest time by age.

**Figure 2:**
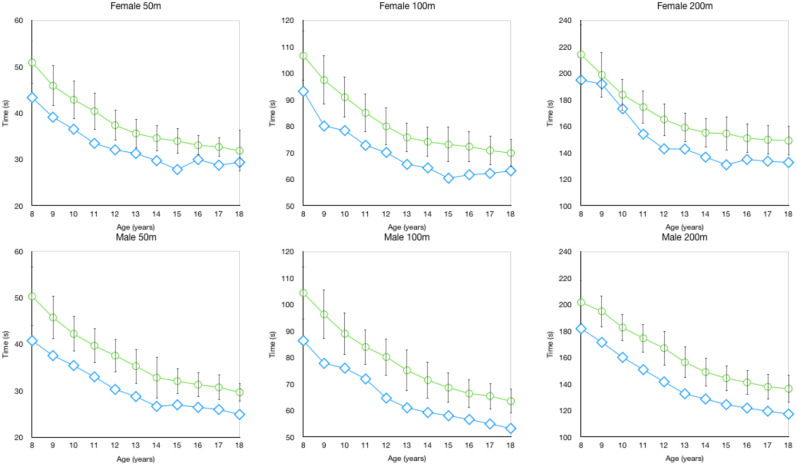
Long course backstroke mean (±SD) race time (s) compared to the overall fastest time by age.

Interestingly, only females had a mean yearly performance decrement (females −0.1% ± at age 18) which occurred in the 200 m short-course event. In the other distances for both short (see Figure 1) and long-course (see Figure 2) there were no differences between genders observed for the yearly percentage improvement in backstroke swimming. Interestingly, Vavrek et al. (2012) also found that in the past 50 years there were no gender differences in the magnitude of relative improvements for freestyle age group swimming performance. It should be clarified that the large standard deviation values in our study demonstrate that across all years, distances and genders there are a considerable number of swimmers that will not improve during a particular age.

To the best of our knowledge, our study is the first study to investigate the variability and progression of adolescent backstroke performance of male and female swimmers. One limitation of our data is that swimmers that swim more often for a particular event have greater opportunity to improve their time across the year, therefore these swimmers could unfairly bias the reported rate of improvement. With this in mind, our data set highlights that swimmers will tend to decrease their performance times by ~1/3 with a decade of backstroke swimming training; how much of this can be attributed to maturation and growth as opposed to training *per se* requires further study. Across the early competitive swimming years (ages 8–10 years old) it is not uncommon to improve close to 10% in a year. However, during the middle years (ages 11–14 years old) an improvement of ~5% is more realistic. While in the later years (15–18 years old) improvements of only 1–2% are to be anticipated.

The percentage improvement for both females and males was higher in short course compared to long course events. The greater number of turns and push-offs made for any given swim distance in short course leads to a redistribution of muscular load and increases the propulsion while providing moderate exercise recuperation. There are several physiological and biomechanical differences between short and long course events including a reduced heart rate and blood lactate concentration in short course events (Telford et al., 1988; Blanksby, 1999). According to Beunen and Malina (1988), any performance improvement comes from biomechanical or biological domains and the increased rate of performance progress of athletes, including swimmers during adolescence, is often due to their anthropometric and physiological maturity.

Practical applications of this data come from coaches attempting to ethically fast-track performance changes through training methods and/or technique interventions. Estimates of the smallest worthwhile yearly change in performance can be determined from an analysis of the year-to-year performance that we have undertaken. Knowledge of the likely mean and range of performance changes across a certain time duration allows coaches to objectively assess the effectiveness of their coaching strategies, while also helping to define realistic future training goals (Pyne et al., 2004).

## Data Availability Statement

The datasets generated for this study are available on request to the corresponding author.

## Ethics Statement

Ethical review and approval was not required for the study on human participants in accordance with the local legislation and institutional requirements. Written informed consent from the participants' legal guardian/next of kin was not required to participate in this study in accordance with the national legislation and the institutional requirements.

## Author Contributions

KA: concept design, data collection, statistical analysis, first draft, and manuscript preparation. CP: concept design, statistical checking, and proof reading. JC: concept design and final proof reading.

## Conflict of Interest

The authors declare that the research was conducted in the absence of any commercial or financial relationships that could be construed as a potential conflict of interest.

## Footnotes

^1^*Swimming New Zealand website*. https://swimming.org.nz/database

## References

[B1] Allen S. Vandenbogaerde T. Hopkins W. (2014). Career performance trajectories of olympic swimmers: benchmarks for talent development. Eur. J. Sport Sci. 14, 643–651. 10.1080/17461391.2014.893020 24597644

[B2] Beunen G. Malina R. M. (1988). Growth and physical performance relative to the timing of the adolescent spurt. Exer. Sport Sci. Rev. 16, 503–540. 10.1249/00003677-198800160-00018 3292266

[B3] Blanksby B. (1999). Gaining on Turns. Paper Presented at the XVII International Symposium on Biomechanics in Sports. Perth: Edith Cowan University.

[B4] Clephas C. Wilhelm A. (2019). Variability of competition results during one season in swimming. Ger. J. Exerc. Sport Res. 49, 20–26. 10.1007/s12662-018-0563-7

[B5] Costa M. Marinho D. Bragada J. Silva A. Barbosa T. (2011). Stability of elite freestyle performance from childhood to adulthood. J. Sports Sci. 29, 1183–1189. 10.1080/02640414.2011.587196 21777055

[B6] Costa M. Marinho D. Reis V. Silva A. Bragada J. Barbosa T. (2010). Stability and prediction of 100-m breaststroke performance during the elite swimmer's career," in *Proceedings of the XIth International Symposium on Biomechanics and Medicine in Swimming* eds P. L. Kjendlie, R. K. Stallman, and J. Cabri (Oslo: Norwegian School of Sport Science), 272–273.

[B7] Fulton S. K. Pyne D. Hopkins W. Burkett B. (2009). Variability and progression in competitive performance of paralympic swimmers. J. Sports Sci. 27, 535–539. 10.1080/02640410802641418 19219736

[B8] Hopkins W. G. (2004). How to interpret changes in an athletic performance test. Sport Sci. 8, 1–7. Available online at: http://sportsci.org/jour/04/wghtests.htm

[B9] Hopkins W. G. Hawley J. A. Burke L. M. (1999). Design and analysis of research on sport performance enhancement. Med. Sci. Sports Exerc. 31, 472–485. 10.1097/00005768-199903000-00018 10188754

[B10] Malina R. (2001). Adherence to physical activity from childhood to adulthood: aperspective from tracking studies. Quest 53, 346–355. 10.1080/00336297.2001.10491751

[B11] Pyne D. Trewin C. Hopkins W. (2004). Progression and variability of competitive performance of olympic swimmers. J. Sports Sci. 22, 613–620. 10.1080/02640410310001655822 15370491

[B12] Stewart A. M. Hopkins W. G. (2000). Consistency of swimming performance within and between competitions. Med. Sci. Sports Exerc. 32, 997–1001. 10.1097/00005768-200005000-00018 10795792

[B13] Telford R. D. Hahn A. G. Catchpole E. A. Parker A. R. Sweetenham W. F. Ungerechts B. Parker A. (1988). Postcompetition blood lactate concentration in highly ranked Australian swimmers, in Swimming Science V. (Champaign, IL: Human Kinetics), 277-283.

[B14] Tor E. Pease D. L. Ball K. A. Hopkins W. G. (2014). Monitoring the effect of race-analysis parameters on performance in elite swimmers. Int. J. Sports Physiol. Perform. 9, 633–636. 10.1123/ijspp.2013-0205 24155134

[B15] Vavrek J. Machin R. D. Tanaka H. (2012). Progression of athletic performance in age-group swimmers in the past 50 years. Int. J. Perf. Anal Sport 12, 608–613. 10.1080/24748668.2012.11868622

[B16] Zacca R. Azevedo R. Peterson S. Vilas-Boas J. Pyne D. Castro F.. (2019). Comparison of intermittent and time trial testing in age-group swimmers. J. Strength Cond. Res. 33 801–810. 10.1519/JSC.0000000000002087 28658078

